# Recent Applications of Machine Learning Algorithms for Pesticide Analysis in Food Samples

**DOI:** 10.3390/foods15030415

**Published:** 2026-01-23

**Authors:** Yerkanat Syrgabek, José Bernal, Adrián Fuente-Ballesteros

**Affiliations:** 1 Center of Physical-Chemical Methods of Research and Analysis, Al-Farabi Kazakh National University, Tole bi Avenue 96a, Almaty 050040, Kazakhstan; yerkanat.syrgabek@kaznu.edu.kz; 2 Department of Fundamental Medicine, Faculty of Medicine and Healthcare, Al-Farabi Kazakh National University, Tole bi Avenue 96, Almaty 050040, Kazakhstan; 3 Analytical Chemistry Group (TESEA), I. U. CINQUIMA, Faculty of Sciences, University of Valladolid, 47011 Valladolid, Spain

**Keywords:** artificial intelligence, Machine Learning, food, chromatography, sample preparation, pesticide

## Abstract

Reliable monitoring of pesticide residues is essential for ensuring food safety. Conventional chromatographic and spectrometric techniques remain labor-intensive, time-consuming, and costly. Recent progress in Machine Learning (ML) provides computational tools that improve the precision and efficiency of pesticide residue detection in diverse food matrices. This review presents a comprehensive analysis of current ML-based approaches for pesticide analysis, with particular attention to supervised learning algorithms such as support vector machines, random forests, boosting methods, and deep neural networks. These models have been integrated with chromatographic, spectroscopic, and electrochemical platforms to achieve enhanced signal interpretation and more reliable prediction from existing analytical data, and more robust data processing in complex food systems. The review also discusses methodologies for feature extraction, model validation, and the management of heterogeneous datasets, while examining ongoing challenges that include limited training data, matrix variability, and regulatory constraints. Emerging advances in deep learning architectures, transfer learning strategies, and portable sensing technologies are expected to support the development of real-time, field-ready monitoring systems. The findings highlight the potential of ML to advance food quality assurance and strengthen public health protection through more efficient and accurate pesticide residue detection.

## 1. Introduction

Food safety is a cornerstone of public health protection, economic prosperity, and sustainable environmental management. For this reason, accurate monitoring of agricultural products has become a global priority [1,2]. Modern agriculture widely uses chemical pesticides such as insecticides, fungicides, and herbicides to reduce crop losses and to improve both yield and quality [1,3,4,5,6,7,8]. However, the excessive or inappropriate use of these chemicals results in the accumulation of harmful residues in food, water, and the surrounding ecosystems [3]. Long term exposure to these residues is associated with serious health problems that include cancer, neurological disorders, reproductive complications, and acute poisoning [9]. To address these risks, international bodies such as the European Commission and the World Health Organization have established strict maximum residue limits (MRLs) [10]. These regulations highlight the need for reliable and sensitive methods for pesticide detection [11,12].

For many years, the qualitative and quantitative analysis of pesticide residues has relied on established chromatographic and spectrometric techniques, particularly gas chromatography–mass spectrometry (GC-MS) [13,14,15] and high-performance liquid chromatography (HPLC) methods [16,17] coupled with mass spectrometry and quadrupole time-of-flight mass spectrometry [3,18]. The aforementioned conventional methods provide high sensitivity and strong multi-residue detection performance [19]. Despite their accuracy, several operational limitations continue to affect their routine use. The procedures often require complex and time-consuming sample preparation steps that involve extraction and purification, and they depend on costly laboratory instruments and trained personnel [1,6,20,21,22]. Most traditional workflows are also sample destructive, which restricts later product sale and reduces opportunities for noninvasive and in situ sampling [20]. Another difficulty concerns the detection of unknown or unexpected contaminants and their transformation products [23]. Traditional targeted approaches are not suitable for this task, although new computational tools such as quantitative structure retention relationships (QSRR) are being developed to predict retention times (RTs) in methods such as liquid chromatography high-resolution mass spectrometry (LC-HRMS) [4,24].

There is an urgent need for fast, affordable, nondestructive, and portable detection systems that can support effective food safety and quality monitoring [2,6,20,25]. In response to this need, recent research has concentrated on nondestructive analytical techniques [20,21]. Current progress includes the development of spectroscopic methods such as ultraviolet (UV), visible-near-infrared (Vis-NIR), and fluorescence spectroscopy [7,19,20,26], together with biosensors [19], electronic noses [27], and highly sensitive surface-enhanced Raman scattering (SERS) platforms [12,28].

Vibrational spectroscopy, particularly Raman and SERS, is recognized for its ability to provide distinct molecular fingerprints, strong sensitivity, and rapid, label-free analysis. These methods can achieve very low detection limits, such as 50 pM for thiabendazole [6,12,28]. Hyperspectral imaging (HSI) combines spectroscopy with imaging and supports fast and nondestructive assessment [29]. It also enables the mapping of residue distribution on food surfaces, including grapes and Hami melons [11,19,30]. New noninvasive sampling methods have been introduced as well. Functionalized strips coupled with GC-MS for adsorbing small molecules from surfaces have been successfully used for apple cultivar authentication and pesticide residue analysis [21]. Electrochemical biosensors and colorimetric strips are also being incorporated into portable devices to allow quick on-site screening [1,25].

A major challenge in adopting these analytical tools is the management and interpretation of the large, high-dimensional, and often complex datasets they generate [11,19,28,30]. To address this issue, the use of machine learning (ML) and deep learning (DL) algorithms has become increasingly effective [21]. ML methods provide strong computational capabilities for data with considerable dimensionality and redundancy. They support automated learning, complex pattern recognition, and more accurate prediction, particularly when identifying mixed or trace contaminants in complex food matrices [11]. These techniques strengthen data interpretation, support nondestructive screening, and enhance quantification in complex food matrices. For classification and quantitative analysis, models such as convolutional neural networks (CNNs) [12,20,28,30], random forest (RF) [2,3,12,19,24,31,32], support vector machines (SVMs) [12], extreme gradient boosting (XGBoost) [11,24,33], and feedforward neural networks (FNNs) have been widely applied. These algorithms deliver very high classification accuracy. For example, a CNN reached 100% accuracy when identifying ten pesticides on pericarps using SERS [28], and RF successfully distinguished apple cultivars from noninvasive molecular data with 100% external validation accuracy [21].

Quantitative prediction has also improved. Malathion detection in sorghum using a stacking ensemble learning model achieved an R^2^ of 0.9798 [11], while decision tree (DT) and gradient-boosting decision tree (GBDT) models produced an average accuracy of 0.953 for estimating organophosphorus pesticide levels with enzyme sensors [31]. A smartphone-based FNN system further demonstrated strong predictive capability for imidacloprid concentration, reaching a residual prediction deviation of 14.75 [34].

At the same time, ML-enhanced platforms support fast and nondestructive sensing for field applications and address the destructive and time-intensive limitations of conventional methods [20,21]. Examples include handheld spectroscopy paired with a 1D ResNet model for detecting pesticide residues on kumquat surfaces with 97% accuracy [19], and on Hami melons using 1D CNN models with 94.00% accuracy [30]. SERS-based systems benefit as well. Combining SERS with CNNs enabled accurate multi-pesticide classification on apple pericarps, and k-nearest neighbors (KNN) and RF algorithms reached more than 97% accuracy for thiabendazole classification in tea samples [28].

Beyond studies focused on pesticide residues, previous research has widely applied ML to food authenticity and geographical traceability. These studies use multi-elemental and isotopic analyses to verify the protected designation of origin claims for high-value products such as olive oil, honey, and wine [35,36,37]. ML has also been applied to quality evaluation and shelf-life prediction. For example, CNNs have been used to automatically assess the ripeness of bananas and tomatoes, while support vector regression has been used to predict the freshness of perishable foods such as salmon filets and beef [38]. Major progress has also been reported in the detection of biological and physical hazards, including foodborne pathogens such as *Salmonella* and *E. coli*, heavy metal contamination by lead, cadmium, and mercury, and mycotoxin levels in grains [37,39]. In addition, ML plays a key role in identifying food fraud and deliberate adulteration, including the detection of Sudan dyes in spices, melamine in milk, and the dilution of premium oils with lower-cost alternatives [40,41]. In the broader field of precision agriculture, AI-based systems are used to optimize irrigation management, predict crop yields using satellite imagery, and monitor livestock health through wearable sensor technologies [42].

This review evaluates recent advances in ML algorithms for pesticide analysis in food matrices. It covers approaches ranging from classical models to DL frameworks applied to spectroscopic, chromatographic, and sensor-based analytical systems. The review emphasizes the specific analytical challenges addressed by analytical methods, including high-dimensional data interpretation, quantitative modeling in complex food matrices, non-destructive analysis, and rapid decision-making for food safety assessment. The review further discusses how ML is applied across analytical workflows, from data preprocessing and feature extraction to model validation and interpretation, while explicitly considering data types, dataset size, and feature dimensionality. By linking data characteristics to model selection, this work highlights how ML provides analytical insight beyond conventional chemometric approaches. This review clarifies when and why machine-learning-based methods offer measurable analytical advantages and how they can be effectively implemented to support pesticide residue analysis and regulatory compliance.

This review is structured as follows. Section 2 outlines the literature search strategy and the criteria used to select relevant studies. Section 3 summarizes the ML methods applied to pesticide residue analysis, with a focus on their analytical functions and limitations. Section 4 examines the use of these methods across different analytical platforms, including spectroscopic, chromatographic, and sensor-based systems. Section 5 addresses recent developments, key challenges, and future research directions, particularly related to data availability, model robustness, and practical implementation. Finally, Section 6 presents the main conclusions and discusses their relevance to food safety monitoring and regulatory compliance.

## 2. Methodology of Literature Review

### 2.1. Bibliometric Analysis

A comprehensive literature search was conducted of the Title, Abstract, and Keywords fields in scientific databases such as Scopus, Web of Science, and Google Scholar. The search strategy used combinations of the following keywords: “machine learning” OR “artificial intelligence” AND “pesticide” AND “food” AND “analysis”. This work was conducted as a narrative systematic review due to the high methodological heterogeneity among the included studies. This heterogeneity included differences in analytical platforms, food matrices, target pesticides, and ML models. To reduce selection bias, predefined inclusion criteria were applied. These criteria included peer reviewed journal articles published in English between 2020 and 2025 that addressed ML applications relevant to pesticide residue analysis in food samples. Studies were excluded if they lacked experimental validation, focused only on non-food matrices, or did not involve analytical data. Title and abstract screening were performed independently, followed by full text evaluation to confirm study relevance.

In this paper, the most frequently analyzed pesticides belong mainly to high-risk groups such as organophosphates and neonicotinoids. The organophosphate insecticides malathion [21,31] and chlorpyrifos, including chlorpyrifos methyl, remain primary targets because of their extensive use and known toxicity. Within the neonicotinoid group, imidacloprid, acetamiprid, and clothianidin are often investigated, especially in platforms designed for simultaneous detection [34]. Important fungicides such as thiabendazole [28] and difenoconazole [20] are also commonly studied. The pyrethroid cypermethrin, including beta cypermethrin and deltamethrin, is another frequent target, together with the widely regulated herbicide glyphosate [28]. Research efforts therefore concentrate on matrices suitable for nondestructive analysis. Apples are often selected as a major matrix for surface residue studies. Grapes [19] and small fruits such as kumquats [20] are also widely examined using HSI or handheld spectrometers. Leafy vegetables like lettuce serve as key models for mixed pesticide quantification, and grains such as sorghum are used for malathion analysis. More complex matrices, including red wine for thiabendazole and fuberidazole detection and milk for SERS-based screening, indicate a growing research scope. Methodologically, recent work has focused on advanced ML and DL techniques to process high-dimensional spectral and impedance data. CNNs, especially 1D CNN models, are frequently reported for classifying spectroscopic data across a range of matrices, including Hami melons, kumquats, apples, and SERS datasets. These models often achieve high accuracy, in several cases approaching 100% [28]. Residual networks (ResNets), and particularly 1D ResNet, are commonly used in handheld and field-deployable systems because of their reported high predictive accuracy. For quantitative prediction and interpretation of complex datasets, ensemble models such as RF are often applied both for classification and for feature extraction. XGBoost is widely chosen for efficient quantitative modeling and for supporting causal inference studies of pesticide removal [33].

### 2.2. Data Synthesis

**Figure 1:**
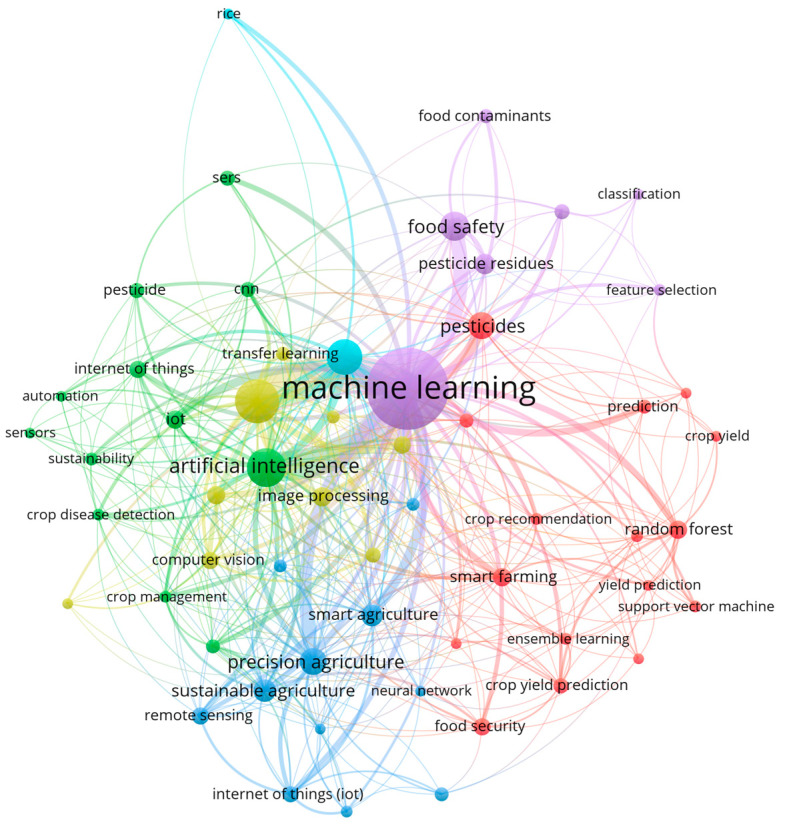
Bibliometric map of author keywords from VOSviewer.

Given the heterogeneity in the target analytes, sample types, and ML methods, the studies were organized into three groups. The first group focused on target pesticides, including organophosphorus compounds, neonicotinoids, and benzimidazole fungicides. The second group concentrated on sample matrices, with attention to food such as apples, grapes, kumquats, sorghum, and corn. The third group covered ML algorithms. The findings were summarized descriptively with emphasis on analytical performance. Keyword trends were visualized using a VOSviewer (version 1.6.20) bibliometric map (see Figure 1) based on author keywords from the included studies.

Central clusters dominated by terms such as ML, pesticides, and food safety indicate a well-established research core focused on predictive modeling and analytical applications. Nearby clusters related to classification, feature selection, and commonly used algorithms, such as RF and support vector machines, reflect a consolidated methodological framework in laboratory-based pesticide analysis. In contrast, peripheral clusters linked to transfer learning, internet of things, portable sensors, and precision agriculture represent emerging research areas with weaker connections to the main research core. This separation points to existing gaps, especially in applying advanced machine learning methods and multi-source sensing systems to standardized and field-deployable pesticide monitoring. These findings indicate that future research should prioritize linking these areas through data fusion, improved model transferability, and application-oriented validation.

## 3. Common Machine Learning Algorithms Used for Pesticide Analysis in Food Samples

### 3.1. Machine Learning Basics

The integration of ML into pesticide detection addresses several limitations of traditional chromatographic methods, including destructive sampling, high operational costs, and long analysis times [43]. In pesticide residue analysis, ML serves a specific analytical role by interpreting high-dimensional and often redundant data generated by techniques such as HSI, SERS, and electrochemical sensors [44,45]. These techniques produce complex signals in which pesticide-related information is frequently masked by matrix effects caused by components such as polyphenols in tea, proteins in milk, or pigments in fruit tissues [46]. Advanced ML models, particularly DL approaches such as one-dimensional CNNs and ResNet, are used to automate feature extraction and to separate overlapping spectral signals. This capability enables accurate identification of pesticide residues and supports detection limits that meet or exceed established MRLs [47,48]. In addition, ML-based nonlinear regression models can handle variable response patterns from mixed pesticide residues, such as combinations of organophosphorus and neonicotinoid compounds, by linking complex spectral variations to precise concentration values [29,49].

For suspect screening applications, ML algorithms including XGBoost and RF are applied to predict RTs and fragment ions in LC-HRMS. This approach is particularly valuable when reference standards for pesticide transformation products are not available. To improve model transparency and regulatory acceptance, visualization methods such as gradient-weighted class activation mapping and saliency maps are increasingly used to identify the spectral regions that drive classification outcomes [21,24]. This ensures that the identification of compounds such as malathion, chlorpyrifos, and thiabendazole is based on meaningful molecular information rather than background noise [21]. A summary of ML algorithms and food matrices is provided in Table 1.

### 3.2. Classification vs. Regression

In pesticide detection, ML models are commonly divided into classification and regression approaches to meet different analytical and regulatory needs [27,51]. Classification models are used to identify pesticide classes such as organophosphorus, carbamates, and neonicotinoids [21], and to perform binary safe or unsafe screening based on MRLs. Common classification algorithms include RF [27], SVM [20], CNNs [30], and specialized techniques like linear discriminant analysis (LDA) [31]. Advanced models including 1D ResNet and 1D CNN have achieved very high classification accuracy, approaching 100%, even when pesticide signals are masked by complex matrix effects in foods such as apples [21], kumquats [20], and Hami melons [30]. In contrast, regression models are essential for the precise quantification of pesticide residues in mg/kg and for predicting chromatographic RTs, which is required for the high-throughput suspect screening of unknown pesticide transformation products [31]. Algorithms such as XGBoost [24], categorical boosting (CatBoost), and stacked ensemble learning are used to model nonlinear relationships in high-dimensional spectral data. These models effectively address challenges such as spectral overlaps in apple juice, matrix interference from proteins in milk, and polyphenols in tea. Classification enables the rapid identification of pesticides in complex samples, while regression supports accurate concentration measurement and the evaluation of pesticide removal during food processing. Together, these approaches form a scalable framework for real-time food safety monitoring [19].

### 3.3. Supervised vs. Unsupervised Learning

**Figure 2:**
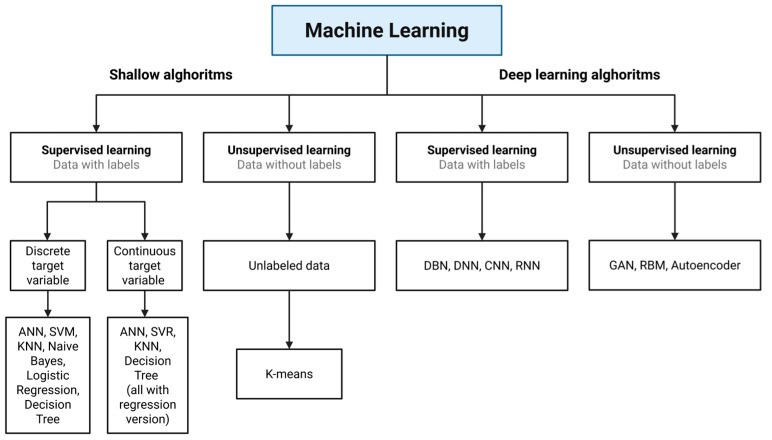
Overview of machine learning categories and algorithms.

ML algorithms are generally divided into two main types, distinguishing between shallow and DL approaches (see Figure 2). In supervised learning, models are trained using labeled data and can handle either discrete target variables, for example, through an artificial neural network (ANN), SVM, KNN, Naïve Bayes, logistic regression, and DT or continuous target variables, using regression versions of these algorithms. Supervised learning methods, including classical algorithms such as RF and support vector machines (SVM), as well as DL models such as 1D ResNet, are widely used for pesticide classification on complex food surfaces. These models enable the discrimination of residues such as prochloraz and cypermethrin on kumquats, with reported accuracies reaching 97% [27]. At the same time, supervised regression models, including GBDT and stacked ensemble learning, are used for accurate residue quantification. These models allow the precise measurement of compounds such as malathion in sorghum grains and enable the prediction of chromatographic RTs, which supports the high-throughput suspect screening of unknown transformation products in LC-HRMS [11].

In contrast, unsupervised learning methods are mainly used for exploratory analysis and for reducing data complexity in techniques such as HSI and SERS. Methods such as principal component analysis (PCA) and t-distributed stochastic neighbor embedding (t-SNE) help visualize clustering patterns and separate pesticide-related signals from background effects, including fluorescence and signal overlap, which are common in matrices such as red wine and tea [54]. While supervised DL models such as 1D CNN improve prediction performance by automatically extracting relevant spectral features without manual wavelength selection, unsupervised approaches such as hierarchical cluster analysis (HCA) support the identification of structural patterns among large numbers of emerging contaminants. The combined use of supervised and unsupervised learning methods converts complex sensor data into reliable information for food safety monitoring and offers an effective alternative to traditional destructive analytical techniques.

Classification predicts discrete categories for tasks, such as identifying food origins. Regression predicts continuous numerical values for quantitative estimation, such as determining chemical concentrations. Most predictive models are developed through supervised learning, which uses labeled training data to map inputs to known outputs, as seen in algorithms such as RF, SVM, and neural networks (NNs). In contrast, unsupervised learning works with unlabeled data to identify patterns, structures, or clusters within the dataset, as demonstrated by methods such as PCA for dimensionality reduction.

### 3.4. Main ML Algorithms

#### 3.4.1. Random Forest (RF)

**Figure 3:**
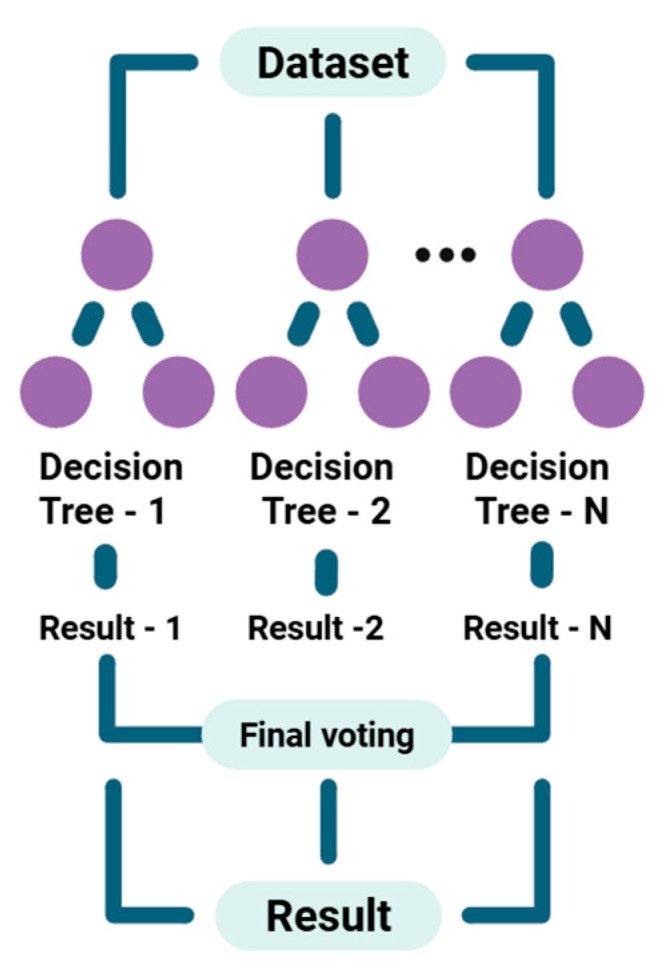
Visualization of random forest algorithms.

The usefulness of the RF algorithm (see Figure 3) in chemical metrology, especially when paired with complex analytical instruments, is applied in multiple studies in the literature [57]. Current research shows that it is an approach used for data interpretation, classification, and prediction in food safety studies [58]. Its high predictive accuracy results from its ensemble design, which enables it to handle the high-dimensional and non-linear data commonly produced by advanced separation techniques and spectroscopic methods.

The RF algorithm has been systematically assessed as a reliable algorithm for predicting chromatographic properties, particularly RT, in LC-HRMS workflows [24]. In studies focused on RT prediction for the suspect screening of pesticides and their transformation products, RF was benchmarked against other DT and NN models, including XGBoost, light gradient-boosting machine (LightGBM), and Keras. The results show that RF reached comparable accuracy to these algorithms and achieved its best performance when built with a full set of chemical descriptors (CDs) [24]. RF accurately predicted RT for more than 94% of pesticides in the validation set, with a RT deviation below 1.0 min. RF, XGBoost, and LightGBM produced similar and highly satisfactory prediction profiles, while the NN model (Keras) performed less effectively. This outcome confirms that RF can capture complex, non-linear patterns typical of QSRR models developed from LC-HRMS data. The study also integrated the RF outputs into a blended prediction strategy, which generated a more accurate and harmonized RT estimate than any individual model alone.

RF is widely used for key classification tasks based on spectroscopic fingerprints because it can process high-dimensional data from optical sensors [6,30]. In Raman spectroscopy, the RF classifier has enabled automated multi-class identification, including the discrimination of 14 pesticide reference samples analyzed with custom-built 785 nm and commercial 532 nm instruments, where it reached 100% accuracy, precision, and sensitivity on the test set [3]. In SERS studies, RF has been compared with algorithms such as KNN, DT, GBDT, and SVC for classifying spectra of several analytes, including thiabendazole contaminants, and achieved accuracies above 97.00% when combined with t-SNE for dimensionality reduction in thiabendazole-contaminated tea samples [32]. RF has also been applied for quantifying melamine and its analogs detected by SERS, where it provided satisfactory fits, though it was ultimately surpassed by a CNN regression model [12]. In HSI research aimed at detecting pesticide residues in agricultural products, RF has been evaluated as a standard classifier; for example, in identifying residue levels in grapes and predicting malathion content in sorghum grains [19]. In a comparative SERS-based classification of ten pesticides detected on pericarp surfaces, RF was tested alongside traditional ML models such as NB, tree-based, LDA, KNN, and SVM, but the CNN approach produced the best performance [28]. RF is also useful for feature optimization; the RF-Recursive Feature Elimination method has been successfully used to select optimal wavelength features for quantifying mixed pesticide residues in lettuce leaves using hyperspectral data [53].

The RF model also supports essential classification and prediction tasks in emerging non-chromatographic analytical platforms [2]. In rapid food authentication, for example, it enabled the correct classification of five apple cultivars using metabolomic fingerprints from two-dimensional gas chromatography–mass spectrometry (GCxGC-MS) data collected through a non-invasive workflow, achieving 100% accuracy [21]. In addition, RF contributed to systems based on time-series impedance data for the fast detection of organophosphorus pesticides, where its combination with LDA produced a model that differentiated dichlorvos, diazinon, and malathion with 96.3% accuracy [31]. Moreover, in food traceability research using high-throughput ambient ionization mass spectrometry, RF outperformed other classifiers such as SVM, Adaboost, and gradient boosting (GB) by achieving 100% accuracy during training and 98.1% in external validation when identifying the production origins of corn samples from their pesticide profiles [2]. Finally, RF was included in a causal inference study evaluating cleaning strategies for removing pyrethroid and carbamate residues from lettuce, although extreme gradient boosting was ultimately identified as the most effective model for that regression task [33].

#### 3.4.2. Partial Least Squares (PLS)

PLS, which includes partial least squares regression (PLSR) for quantitative analysis and partial least squares discriminant analysis (PLS-DA) for classification, is widely recognized as an effective chemometric approach used with many analytical techniques, particularly when handling complex and high-dimensional chemical data. Its main advantage is that it can manage the strong multicollinearity often present in spectral and chromatographic profiles by extracting latent variables that maximize the covariance between the input data and the target response or class variable [26].

In separation science, PLS methods play an important role in converting complex chromatographic patterns into reliable predictions or classifications. For example, PLS-DA has been applied to metabolomic profiles generated from GCxGC-MS data, where it successfully highlighted distinct metabolic signatures linked to different apple cultivars and enabled accurate classification [21]. For quantitative analysis, PLS-R serves as a strong multivariate calibration model, particularly when combined with a gas chromatography–flame ionization detector (GC-FID) and dispersive liquid–liquid microextraction, and it has proven effective for determining pesticide residues such as ametryn and chlorpyrifos in challenging saffron matrices, where it uses detailed GC profiles to predict concentrations more accurately than simple univariate methods. In the demanding application of determining pesticide residues in complex saffron matrices, PLSR utilizes the detailed GC profiles to simultaneously predict pesticide concentrations, confirming its superior performance compared to simpler univariate calibration methods. Moreover, PLS approaches are also used to predict liquid chromatography (LC) RTs, as PLS-R supports QSRR modeling alongside linear regression (LR), multiple linear regression, support vector machine regression (SVMR), and multilayer perceptron regression. Although non-linear models often perform better, PLS-R continues to serve as a fundamental tool for examining structure–retention relationships [4].

PLS methods are widely applied across many spectroscopic techniques used for non-destructive analysis and quality control, since they help simplify high-dimensional spectral data and extract the most relevant information. In SERS studies, for example, PLS is used to reduce dimensionality before ML classification is performed on complex spectral fingerprints [32]. In HSI research aimed at detecting pesticide residues in crops such as grapes [19], PLS-DA functions as a standard classification approach, while quantitative HSI studies often rely on PLSR and modified PLS models to measure residue concentrations; these models have shown strong predictive ability for compounds such as chlordecone in soil, mixtures of 2,4-dichlorophenoxyacetic acid, chlorpyrifos, cypermethrin, and carbaryl in cocoa beans, and triadimefon in propolis [11]. When fluorescence spectroscopy is combined with chemometrics to evaluate chemical content, PLS-R is frequently used as a benchmark because it reduces the high dimensionality of spectral data while retaining essential variables, as shown in studies modeling difenoconazole levels in cabbage, where PLSR was compared with broad learning system and echo state network (ESN) algorithms [26]. In addition, Raman spectroscopy applications use PLS regression for detecting individual compounds such as pyrimethanil in apples and multi-label PLS-DA for classifying complex mixtures, where it maximizes class separation in noisy datasets, even though DL methods may achieve lower classification error in some cases [6].

#### 3.4.3. Support Vector Machine (SVM)

**Figure 4:**
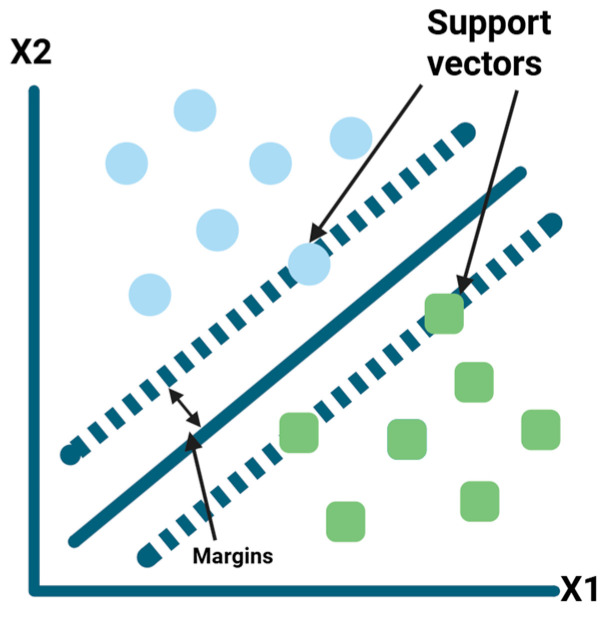
Visualization of support vector machine algorithms.

SVM method (see Figure 4) kernel-based approaches are appreciated because they can model the complex non-linear patterns that commonly appear in spectroscopic and chromatographic data, and they therefore offer reliable options for both classification and quantitative prediction [4,19].

In spectroscopic classification, SVM has been widely tested and compared across many applications, and although it performs reliably, it is often outpaced by more advanced DL models. For example, in HSI studies aimed at detecting pesticide residues in grapes, SVM functioned as a standard supervised recognition method and produced acceptable analytical performance within reported validation ranges. However, ResNet models generally achieved higher accuracy because they could extract more detailed and complex features [19]. A similar trend appeared in handheld visible and near-infrared (VNIR) spectroscopy for classifying pesticide residues on kumquat surfaces, where the hybrid SPA-SVM approach was applied, yet both 1D convolutional neural network (1D-CNN) and 1D residual network (1D-ResNet) models clearly performed better, showing that DL architectures are often more capable of capturing subtle spectral variations essential for precise classification [20].

The use of SVM in combination with Raman spectroscopy, especially Surface-Enhanced Raman Spectroscopy, shows how versatile this method can be across different analytical tasks. In one study, SVM was compared with six other algorithms, including Naïve Bayes, Tree, LDA, RF, k-nearest neighbors, and a CNN for classifying ten pesticide types on pericarp samples, and it achieved nearly 100% accuracy in both the training and internal validation sets [28]. In another application focused on detecting melamine and its analogs in milk using SERS, a PCA-SVM model reached a high accuracy of 92.25%, as PCA reduced noise and dimensionality before SVM classification [12]. SVM has also been adapted for multi-label classification, such as identifying up to nine pesticide compounds in apple mixtures, where it was implemented through a MultiOutputClassifier to address each pesticide label independently; in this context, it performed competitively and outperformed other models for specific compounds like trifloxystrobin and spirotetramat [6].

Beyond optical and vibrational spectroscopy, SVM also plays an important role in systems developed for rapid screening. In electronic nose applications designed to detect alpha-cypermethrin residues on Swiss chard, SVM provided a quantitative decision rule and achieved a classification success rate of about 92.3% after PCA was used for preliminary exploratory analysis [27]. In addition, studies using LC-HRMS to build classification models from MS fragmentation spectra of chemical contaminants evaluated SVM alongside ANNs, RF, and XGBoost. Although SVM showed strong recall, reaching 0.823, it was generally ranked below the ANN model, which delivered higher accuracy, precision, and F1 scores, indicating that SVM remains competitive yet is sometimes surpassed by more flexible NN architectures [27].

In quantitative analysis, SVR is highly effective and has been applied to several challenging prediction tasks. For example, SVR was used for the simultaneous quantification of thiabendazole and fuberidazole in red wine using second-derivative constant-energy synchronous fluorescence spectroscopy, where it consistently produced the lowest root mean square error (RMSE) values compared with linear regression, Gaussian regression, DTs, and NN models across both extraction and dilution procedures [5]. A related approach, PCA-SVR, has also been applied to predict melamine concentrations from SERS data in milk, with PCA reducing noise and dimensionality before SVR modeling [12]. In chromatography, SVR remains a strong option for predictive modeling, as SVMR has been cited in QSRR studies as a powerful nonlinear method for estimating pesticide RTs from LC data, often performing competitively with other advanced algorithms [4].

Although SVM methods are highly capable, especially for modeling non-linear data, they do not always match the performance of advanced ensemble or DL approaches. In traceability studies that classified the geographical origin of corn using ambient ionization mass spectrometry data, for example, the RF model showed markedly higher accuracy and greater robustness than SVM [2]. A similar pattern was observed in a HSI study evaluating pesticide levels in sorghum, where least squares support vector regression (LSSVR) was described as a traditional method that often produced lower predictive accuracy when handling high-dimensional HSI data compared with newer ensemble techniques [11]. Even so, the continued development and successful use of SVM, SVR, and their hybrid models demonstrate their lasting relevance in analytical chemistry, as they provide a reliable, well-established baseline and comparative tools for addressing complex analytical problems.

#### 3.4.4. K-Nearest Neighbors (KNN)

**Figure 5:**
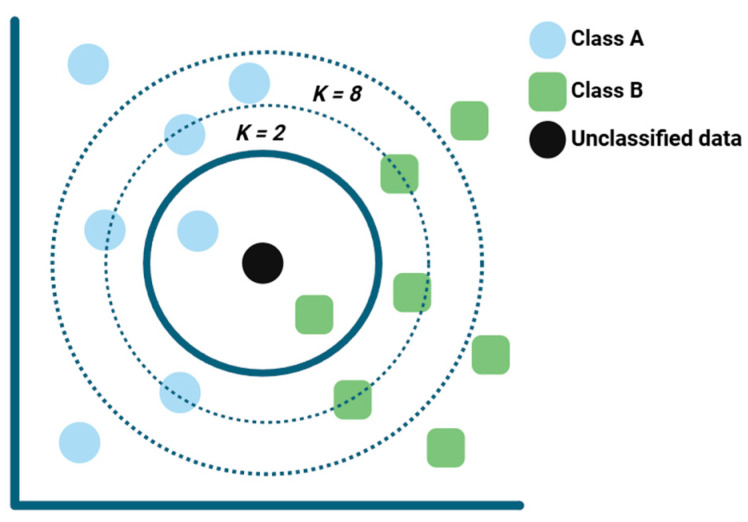
Visualization of k-nearest neighbor algorithms.

The KNN algorithm is a basic non-parametric method appreciated for its simplicity in both classification and regression (see Figure 5), as it recognizes patterns by measuring distances between samples in the feature space.

The flexibility of the KNN algorithm, used both for classification and regression, is evident across several analytical platforms that handle high-dimensional data, including spectroscopy and electronic sensing. In HSI studies aimed at rapid, non-destructive pesticide residue detection, KNN often serves as a baseline model for comparison. For example, in detecting various pesticide residues on Hami melon surfaces using HSI, KNN achieved an accuracy above 80%, although a one-dimensional CNN later produced more reliable results [30]. Likewise, in HSI analysis of pesticide residue levels in grapes, KNN was included among the conventional ML classifiers, highlighting its continued role as a reference method in evaluating performance within complex spectral classification workflows [19].

KNN has also been applied beyond optical spectral methods, particularly in systems that generate time-series data from integrated sensors. In one study introducing a high-throughput platform for the rapid quantitative detection of organophosphorus pesticide residues, the KNN regressor was used alongside RF and XGBoost to predict the removal of pyrethroids and carbamates from lettuce, showing its usefulness for causal inference based on immersion cleaning conditions [33]. In fluorescence spectroscopy aimed at assessing difenoconazole content in cabbage, KNN was used to build a qualification model to determine whether residue levels exceeded regulatory limits, emphasizing its value as a foundational tool for preliminary safety evaluations based on spectral fingerprints [26].

In studies using Surface-Enhanced Raman Spectroscopy for highly sensitive pesticide detection, the KNN algorithm is often included in comparative evaluations to assess its performance in multi-class classification settings. For example, when classifying ten pesticide types on pericarp samples, KNN was benchmarked alongside Naïve Bayes, DTs, LDA, SVM, RF, and a CNN. Although the CNN delivered near-perfect accuracy, the presence of KNN helped establish the baseline performance achievable with traditional ML approaches [28]. A similar pattern emerged when classifying SERS spectra from six food-safety-related molecules, including thiabendazole, where KNN was evaluated among DT, RF, GBDT, and SVC models; in this case, KNN showed the most stable performance across different dimensionality reduction techniques, indicating that it can remain reliable when data characteristics vary or contain uncertainty [32]. Since KNN relies on distance-based metrics, its effectiveness depends on appropriate parameter optimization, particularly the careful selection of neighborhood size, which is essential for achieving strong classification outcomes [26].

#### 3.4.5. Neural Networks (NN)

##### Neural Networks in Spectroscopy and Imaging

**Figure 6:**
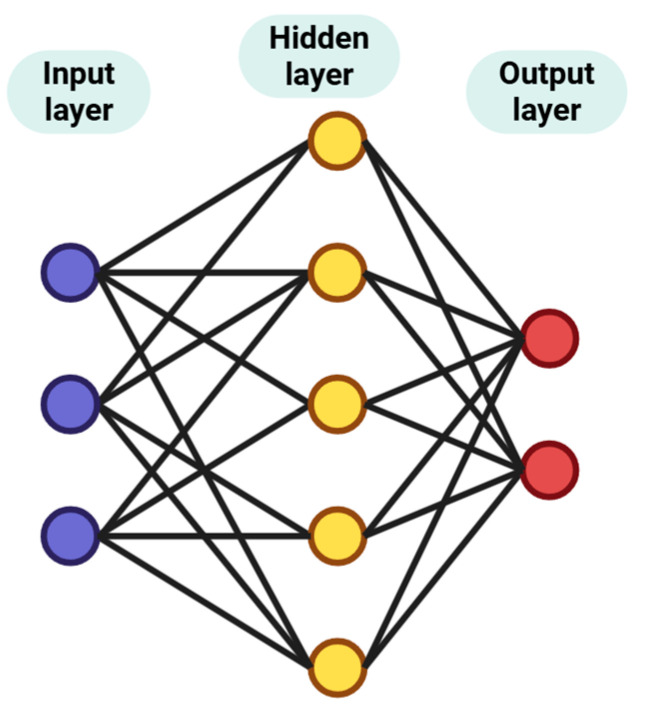
Visualization of neural network algorithms.

NN models, including FNNs, ANNs, CNNs, and Residual Neural Networks (see Figure 6), are widely applied especially in advanced spectroscopic and chromatographic analysis for safety monitoring. These models are used because they can identify complex and non-linear patterns in high-dimensional chemical data. In many cases, they perform more effectively than traditional ML methods.

NNs are mostly applied together with spectroscopic techniques, where they support both classification and quantitative prediction based on characteristic spectral fingerprints. In hyperspectral imaging, which produces large and often redundant datasets, DL models such as CNNs and residual neural networks (ResNet) consistently show better performance than conventional algorithms. In detecting pesticide residues in grapes, both CNN and ResNet models produced strong results, with ResNet reaching the highest accuracy for Vis-NIR spectra, above 93%, and performing at a similar level to Logistic Regression and SVM models for NIR spectra, achieving accuracy above 95% [20]. ResNet is particularly suitable because its skip connections reduce the degradation problem that affects deep networks, which supports stable training. A similar trend was observed for detecting pesticide residues on Hami melon surfaces using hyperspectral imaging, where a multi-branch one-dimensional CNN with an attention mechanism reached higher accuracy than k-nearest neighbor and RF models, both of which exceeded 80% accuracy [30]. The enhanced 1D-CNN structure, often composed of three parallel branches and residual blocks, strengthens robustness and non-linear representation and reduces issues such as gradient loss that occur in simpler networks. DL’s advantage is further confirmed in the rapid and non-destructive detection of pesticide residues on kumquat surfaces using a handheld Visible and Near-Infrared spectrometer, where the 1D-ResNet model achieved 97% overall accuracy and clearly outperformed the combined SPA-SVM method [20].

The quantitative estimation of malathion in sorghum grains used an advanced Stacked ensemble learning model that combined several base learners, including gradient-boosting methods [11]. Although gradient-boosting DTs such as GBDT, XGBoost, LightGBM, and CatBoost are not NNs, they function as powerful non-linear predictive tools and are often compared with NN architectures in advanced ML studies [24]. NN applications are also evident in fluorescence spectroscopy for pesticide analysis. In a study on difenoconazole content in cabbage, the broad learning system, which is a NN designed to handle high-dimensional data efficiently, was compared with PLSR and ESN [15]. The broad learning system produced the best performance, with correlation coefficients above 0.90 for content prediction [26].

##### Neural Networks in Chromatography and Mass Spectrometry

NN models play an important role in predictive modeling in analytical chemistry and are also applied to chromatographic behavior. In HPLC, ML is used to predict chromatographic RT, which is essential for suspect screening of pesticides and their transformation products [24]. NN algorithms implemented through Keras were compared with DT methods such as XGBoost, RF, and LightGBM for RT prediction [28]. DT models performed better when using comprehensive chemical descriptors, while the Keras model achieved the best results when trained on molecular fingerprints, with 90% of pesticides showing a RT deviation of less than 1.0 min [24]. Although the Keras model required careful tuning and specific data preparation, it showed strong potential for handling large datasets but had limited performance on the smaller dataset used in the study compared with DT models trained on their preferred features. A combined prediction strategy that integrated Keras with the tree-based algorithms produced more accurate harmonized RT estimates. Earlier research also used multilayer perceptron regression together with linear models and SVM in QSRR modeling to predict pesticide RTs [4].

ANN have also been used for non-targeted screening of chemical contaminants in LC-HRMS by classifying compounds according to their MS2 spectra [51]. A classification tool named AIHazardsFinder was developed using an ANN and achieved the best performance among the tested models, with an accuracy of 0.869, a precision of 0.902, and an F1 score of 0.839 for identifying 32 contaminant classes, including pesticides, veterinary drugs, and mycotoxins. These results were higher than those obtained with RF, SVM, and XGBoost models [51].

##### Neural Networks in Other Sensor Technologies

The back propagation neural network (BPNN) is a widely used multilayer feedforward model applied in sensor fusion and spectral detection. In a study using ultraviolet spectroscopy to detect three neonicotinoid pesticides (imidacloprid, acetamiprid, and clothianidin) in apple juice, the BPNN model optimized with the sparrow search algorithm showed the highest prediction accuracy and the best fitting performance. Its results were superior to those of the extreme learning machine, the standard BPNN, and the particle swarm optimization–BPNN models [7].

For quantitative prediction using fluorescence signals and a smartphone interface, a FNN model was developed and applied for imidacloprid detection. The model used RGB values from smartphone-captured fluorescence images as input and achieved high predictive accuracy, with an R^2^ greater than 0.9953 on the test set. It also showed strong robustness and performed better than traditional regression models such as DT, SVM, Bayesian linear regression, and ridge regression. The deep neural structure of the FNN helped capture non-linear relationships effectively, resulting in a high residual prediction deviation value of 14.75 [34].

### 3.5. Validation of Machine Learning Models

Systematic validation of ML models is essential to ensure that predictions generalize new and unseen data rather than reflecting noise learned from the training set [54]. This process is based on the bias variance tradeoff, where bias refers to errors caused by overly simple models that underfit the data, and variance refers to errors caused by models that are too sensitive to variations in the training data and overfit. Effective validation identifies an appropriate model complexity that minimizes the combined impact of bias and variance, thereby reducing the risk of learning random patterns.

A core principle of validation is that a model must not be evaluated using the same data on which it was trained. Standard practice involves dividing the dataset into a training set for model fitting, a validation set for parameter tuning and model selection, and an independent test set for unbiased performance evaluation [54]. When data availability is limited, k-fold cross-validation is commonly used so that each data point contributes to both training and validation, which improves the stability of performance estimates. For applications with high regulatory or analytical importance, nested cross-validation is applied to separate model optimization from final performance assessment and to reduce selection bias.

In regression analysis, model performance is evaluated by examining the size and pattern of residuals between predicted and observed continuous values. Mean absolute error provides a stable measure because it applies a linear penalty to all errors and is less affected by outliers. In contrast, mean squared error and RMSE assign greater weight to large errors, which helps identify severe prediction failures. The coefficient of determination, R^2^, indicates how much of the variance in the data is explained by the model. However, this metric can increase simply by adding more variables, even if they are not meaningful. For this reason, adjusted R^2^ is preferred, as it accounts for the number of predictors and penalizes the inclusion of features that do not contribute significantly to model performance [40].

The confusion matrix is the basic tool used to evaluate classification performance. It summarizes the number of true positives, true negatives, false positives, and false negatives produced by a model (see Table 2). Although accuracy, defined as the proportion of correct predictions out of all cases, is easy to understand, it can be misleading when class distributions are imbalanced. In such cases, a model may achieve high accuracy by predicting only the majority class. For this reason, precision and recall are more informative metrics. Precision measures how reliable positive predictions are and is especially important when false positives are costly. Recall measures the ability of a model to correctly identify positive cases and is critical when false negatives carry serious consequences, such as contaminant detection or medical diagnosis.

## 4. Application of ML Models to Pesticide Analysis in Various Analytical Techniques

### 4.1. Gas Chromatography

ML methods integrated with gas chromatography improve the interpretation of complex matrices by supporting both classification and quantitative analysis through different forms of chromatographic data representation. Two main applications illustrate this interaction: high-dimensional metabolomic fingerprinting and chemometric quantification. For apple cultivar authentication, a validated noninvasive workflow used bidimensional gas chromatography coupled with mass spectrometry to produce high-dimensional data. The resulting molecular concentrations and metabolomic fingerprints from the apple surface, representing almost 800 identified molecules such as esters, terpenes, and fatty acids, were used as chemical features and processed with the RF classifier [13]. The study applied a rigorous design by dividing the 44 apple samples into an 80% training set and a 20% external validation set to ensure reliable cultivar classification. In contrast, for the quantitative determination of the pesticides ametryn and chlorpyrifos in saffron, a chemometrics-assisted approach was combined with GC-FID. In this case, pre-processed chromatographic segments and peak areas formed the independent variables, while the known pesticide concentrations formed the dependent variables. PLSR was selected because it is effective for linking complex chromatographic signals with concentration values. The model was built using 24 calibration samples prepared across eight concentration levels ranging from 0.5 to 100 ng mL^−1^, and predictions were then made on spiked saffron samples. GCxGC-MS applies supervised classification through RF to interpret complex metabolomic fingerprints for qualitative discrimination, whereas GC-FID uses multivariate chemometric regression through PLS regression to analyze segmented chromatographic data for precise quantitative determination in complex food matrices [21].

GC-based techniques are considered the gold standard for pesticide residue determination because they provide high sensitivity, accuracy, and confirmatory reliability, particularly for organophosphorus, organochlorine, and pyrethroid compounds [41]. However, these methods are limited by high operational costs, long analytical times, and the need for destructive and labor intensive sample preparation [34,59,60]. The integration of advanced chemometric models and multivariate calibration methods, such as partial least squares regression and central composite design, allows the optimization of key extraction parameters in dispersive liquid–liquid microextraction. This approach improves the detection of target analytes, including ametryn and chlorpyrifos, in complex matrices such as saffron tissues and pericarps. Effective application of ML requires rigorous data inputs, including high-resolution total ion chromatograms, extracted ion chromatograms, or mass spectral data, together with large training datasets that often include more distinct pesticide structures to ensure robust model validation. To preserve analytical reliability, these workflows also require multiple and carefully controlled data pre-processing steps.

### 4.2. Liquid Chromatography

The scientific literature shows that ML combined with LC, especially high-resolution mass spectrometry and tandem mass spectrometry, improves suspect screening and predictive modeling of chemical contaminants because it reduces the need for complex sample pretreatment and long analysis times [30]. These applications rely on two main data types: chemical structure information for predicting retention behavior through quantitative QSRRs, and high-dimensional spectral data such as MS^2^ spectra for classifying and identifying unknown compounds [24,51]. For predictive RT modeling, ML transforms the chemical structures of target compounds, such as 398 pesticides, into numerical features including chemical descriptors and extended connectivity fingerprints often derived from SMILES notation [24]. One study divided 398 pesticides into a training set of 321 and a validation set of 77 to compare algorithms such as XGBoosting, RF, LightGBM, and Keras. DT-based algorithms showed the best performance when using large Chemical Descriptor sets of 3874 features, while the Keras NN performed best with 1024 fingerprint features, with more than 90% of validation pesticides predicted within a RT deviation of less than 1.0 min [24]. For non-targeted screening, ML uses MS^2^ spectra to build classification models. An ANN trained on more than 1600 contaminants across 32 chemical classes used 10,246 MS^2^ spectra and applied 10 rounds of 10-fold cross validation, with training and test sets separated by InChIKey to avoid overlap. This approach allowed effective comparison of ANN, RF, SVM, and XGBoost for classifying unknown spectral features in food samples [51]. RT prediction has also been developed for 823 pesticide residues using the Monte Carlo method with data divided into active training, passive training, calibration, and validation subsets [4]. The aforementioned studies show that ML can use molecular structure data for retention prediction or MS^2^ data for classification, reducing the complexity and time required for contaminant identification compared with conventional LC-based workflows [24,51].

Integrating artificial neural network platforms, such as AIHazardsFinder, helps reduce the LC-HRMS bottleneck by automating the classification of unknown MS^2^ spectra. These systems achieve classification accuracies above 80% and false-positive rates often below 0.2%. In addition, quantitative structure–retention relationship models support in silico prediction of chromatographic RT, which is important for suspect screening of emerging transformation products when experimental standards are not available.

The main advantage of integrating liquid chromatography with ML is the ability to perform in silico screening, which reduces the need for costly chemical standards and shortens method development time. By predicting RTs and comparing them with experimental values, ML models increase confidence in compound identification during suspect screening. In addition, ML can model non-linear relationships between molecular descriptors and chromatographic behavior that are often not captured by traditional quantitative structure–retention relationship models. Despite these advantages, several limitations remain. Model performance depends on the quality and chemical diversity of the training dataset, and models developed using a limited range of compound classes may not perform well for new structures. Another challenge is inter-laboratory shift, where RTs differ across LC systems or column batches. This variability often requires additional recalibration or transfer learning steps to maintain predictive accuracy across different analytical platforms.

### 4.3. Hyperspectral Imaging

The integration of ML algorithms with HSI has become an important approach for non-destructive, rapid, and accurate detection of pesticide residues in agricultural products, mainly because it helps process the large and often redundant data produced by HSI systems [19]. The data used in this field consists primarily of high-dimensional spectral information collected across Vis-NIR and short-wave infrared ranges, which provide detailed chemical and spatial features for model development. These spectral datasets are used as inputs for both conventional ML algorithms such as LR, SVM, and RF, and DL models such as CNN, RNN, and other ensemble architectures. A consistent element in these studies is careful data partitioning. For example, in the qualitative detection of pesticide residues on grapes, models including LR, SVM, RF, CNN, and ResNet achieved strong accuracy, with ResNet exceeding 93% in the Vis NIR range and LR exceeding 97% for NIR data [19]. Likewise, in a quantitative analysis of malathion in sorghum using NIR hyperspectral imaging, a stacking ensemble learning model was trained using an 80% calibration set and a 20% prediction set prepared with the SPXY algorithm [11]. In multi-class pesticide classification on Hami melon surfaces, a multi-branch one-dimensional CNN trained on fused VNIR and short-wave infrared range spectra used 1600 averaged spectral samples divided into training, validation, and test sets in a 5-to-1-to-2 ratio, producing an overall accuracy of 94.00% [30]. In general, DL approaches such as CNN and ResNet outperform conventional ML models because they can extract deeper and more meaningful features from spectral and spatial spectral information, which helps address the high redundancy and reduced accuracy often observed when traditional methods are applied to complex hyperspectral datasets [19].

The main advantage of this technique is its ability to generate chemical maps that show the spatial distribution of compounds across heterogeneous surfaces in real time. This capability supports high-throughput quality control and applications in precision agriculture. However, integration with ML presents some limitations. A challenge is the curse of dimensionality, which arises from the large volume of redundant data collected across hundreds of adjacent spectral bands. This issue requires computationally demanding preprocessing steps, such as multivariate scatter correction or standard normal variate transformation, as well as advanced feature selection methods to identify the most informative wavelengths. Effective hyperspectral imaging and ML modeling depend on strict data quality requirements. These include accurate spectral calibration using black and white reference standards to correct for lighting variation and sensor noise, and reliable ground truth labels. Such reference data are usually obtained from conventional analytical techniques, including LC-MS, and are important for training and validating predictive models such as 1D convolutional neural networks or support vector machines.

### 4.4. Raman and Surface-Enhanced Raman Scattering Spectral Analysis

ML combined with Raman spectroscopy SERS provides a strong analytical approach for managing high-dimensional spectral data, especially in complex food matrices [32]. These applications rely mainly on high quality Raman or SERS spectra, which offer distinct molecular fingerprints for identifying and quantifying analytes such as pesticides, including thiabendazole, chlorpyrifos, dimethoate, and mixtures of multiple pesticides, as well as contaminants like melamine and related compounds [3,6,12,28,32]. Before applying ML, spectra are usually preprocessed through baseline correction, normalization, and dimensionality reduction methods such as t distributed Stochastic Neighbor Embedding or PCA to extract relevant chemical information and improve model performance [6,12,32]. Data partitioning is designed to ensure generalization, and many studies divide hundreds of spectra per analyte into training and testing sets, with about 80% used for training and 20% for external testing [32]. In more complex multi-class problems, such as identifying ten different pesticides on fruit surfaces with CNNs, studies use very large datasets, for example, 5000 Raman map groups, dividing them into training and internal validation sets in a 7-to-3 ratio and using a separate external test set, such as 500 spectra from real samples, to confirm accuracy under realistic conditions [28]. When comparing methods, DL models such as CNNs and RNN generally perform better than traditional ML approaches because they can learn detailed patterns in complex spectral mixtures, often achieving accuracies close to 100% in pesticide and contaminant identification [6,12,28]. In contrast, methods like RF and k-nearest neighbor are commonly paired with dimensionality reduction, for example, t-SNE or PCA combined with SVM, which supports both complex classification tasks and the development of reliable Raman fingerprint libraries for reference compounds [3,12,32].

The main advantage of this combined approach is its ability to achieve highly sensitive, label-free detection, which is enhanced by surface-enhanced Raman scattering hotspots. At the same time, ML algorithms can extract meaningful chemical fingerprints from complex and noisy spectra that cannot be reliably interpreted by manual analysis. This capability enables rapid identification of trace contaminants with minimal sample preparation, making the approach suitable for on-site food safety control and environmental monitoring. Despite these strengths, several limitations remain. Signal reproducibility is affected by variability in SERS substrates, where small differences in nanoparticle distribution can cause inconsistent signal intensities. In addition, strong background fluorescence from organic matrices can mask Raman signals, and structurally similar compounds often generate overlapping spectral features. Resolving these challenges requires advanced DL models and large, chemically diverse training datasets to ensure accurate discrimination.

### 4.5. Smartphone-Based Analysis

The integration of ML with smartphone technology has led to the development of portable analytical systems that enable the rapid and intelligent detection of chemical contaminants by using a smartphone as the main data collection tool. These systems convert visual information recorded by the smartphone camera, usually fluorescence signals or color changes on biosensors or test strips, into quantitative features suitable for ML modeling. For example, in an imidacloprid detection platform, the inputs consisted of RGB values taken directly from fluorescence images captured through a smartphone application with a Color Picker function [34]. This dataset included 880 RGB sets from 220 fluorescence images collected at different concentrations and was modeled using a FNN [34]. The data were divided in a supervised manner, with 70% used for training, 15% for validation, and 15% for testing. Other rapid detection systems follow a similar strategy, using smartphone images of test strips and extracting a multi-color feature index from the RGB channels, which reflects changes in pesticide concentration. These indices are often analyzed using improved regression methods such as an optimized Gaussian process model, which has shown high predictive accuracy with an R^2^ value of 0.935 [1]. In comparison, both types of applications rely on ML to interpret visually derived digital features, either through FNN models for fluorescence-based prediction or optimized regression for color index analysis. These approaches provide an efficient and cost-effective alternative to traditional laboratory instruments, as long as the color data are supported by careful training and validation [1,34].

Lighting variation is a major source of error because changes in ambient illumination directly affect the brightness and contrast of colorimetric and fluorometric signals. In colorimetric test strip analysis, uneven lighting can lead to inconsistent thresholding. This problem is commonly addressed using advanced binarization methods, such as the Otsu algorithm, which selects appropriate global or local thresholds. In fluorescence measurements, external light interference is reduced by using dedicated UV light boxes that provide a stable excitation source. A 302 nm UV lamp is typically applied to ensure that recorded signals reflect the response of the fluorescent probe rather than environmental noise. In addition, smartphone applications that include automatic calibration and one-touch data acquisition improve usability and data consistency in diverse and poorly controlled field conditions [55].

Camera-related differences arise from the varying sensitivities of charge-coupled device and complementary metal oxide semiconductor sensors. These differences introduce hardware-related noise that can distort important spectral information. Common artifacts include salt and pepper noise, Gaussian noise, and Rayleigh noise, which require denoising methods such as median filtering. A 3 × 3 filtering window is commonly used because it reduces noise while preserving image detail. Digital color recognition software converts optical signals into red, green, and blue values, but these channels do not respond equally. Experimental results show that blue and green features in some probe systems are closely clustered, whereas red channel features respond more to changes in pesticide concentration. Therefore, ML models must account for channel-specific behavior to achieve high coefficients of determination and low root mean square error values [55].

The aforementioned method is low in cost, highly portable, and provides very fast results, often within 1 min, which makes it suitable for users with limited technical skills in resource limited settings. However, its performance is influenced by environmental factors such as uneven lighting, variable shooting angles, and differences in camera hardware. These factors may introduce noise and reduce accuracy in quantitative analysis. As a result, this approach is mainly appropriate for rapid, field-based screening of agricultural products, such as rice, ginger, and leafy vegetables, during early production or local inspection stages. Reliable model performance requires careful image preprocessing, including median filtering to reduce noise and Otsu binarization for region segmentation, as well as normalization methods to limit external variability. In addition, advanced ML models, such as feedforward neural networks or genetic-programming-based symbolic regression, are needed to capture the non-linear relationships between color features and pesticide concentrations.

### 4.6. Spectrophotometer-Based Analysis

ML helps resolve the main limitation in spectrophotometer-based analysis, which is the difficulty of separating overlapping spectra in complex matrices [5,7]. These integrated systems rely on high-dimensional optical data. Conventional instruments use UV spectra, such as those obtained from 84 apple juice samples [7], or detailed fluorescence spectra, including second-derivative constant energy synchronous fluorescence spectra for quantifying thiabendazole and fuberidazole in red wine [7], and three-dimensional fluorescence spectra for characterizing difenoconazole in cabbage [26]. In contrast, portable platforms use smartphone or sensor-based images, converting color changes or fluorescence patterns into digital features such as raw RGB values or multi-color feature indices [1,34]. Reliable model development depends on careful data partitioning. UV-based quantitative models, such as SSA-BPNN, use a calibration set for training and a separate prediction set for external evaluation [7]. Studies using synchronous fluorescence frequently apply tenfold cross validation to improve generalization before testing on an independent test set [5]. When comparing techniques, advanced ML approaches such as SVR in synchronous fluorescence analysis or the broad learning system for three-dimensional fluorescence modeling [26] generally achieve higher fitting accuracy and stronger predictive performance than simpler linear or nonlinear regression methods. These results show that ML offers effective solutions for multi-component detection when physical separation is impractical or too slow [1,5].

This approach offers high efficiency and allows rapid analysis of many samples within a short time, often within minutes, without the need for complex separation steps or expensive reagents. The use of smartphone-based platforms combined with ML models such as feedforward neural networks also enables on-site monitoring. This supports the real-time safety assessment of agricultural products, with recovery rates often higher than 95%. However, the method is sensitive to matrix effects, such as background signals from red wine pigments, and to noise in spectral data, which can reduce the accuracy of simple models. To improve reliability, advanced data preprocessing is required, including Savitzky–Golay smoothing and feature selection methods such as uninformative variable elimination or the successive projections algorithm. In addition, models must be carefully validated using dataset splitting and 10-fold cross-validation to ensure stable performance across different samples and environmental conditions.

### 4.7. Other Sensor-Based Analysis

ML integrated with sensor-based analytical systems, including ambient ionization mass spectrometry, electrochemical biosensors, and electronic noses, supports the conversion of raw sensor signals into reliable classification and quantitative outputs for field-oriented and non-traditional applications. Electrochemical enzyme biosensors generate time-series impedance data during enzyme inhibition reactions, and these data serve as features for both pesticide classification and concentration prediction [31]. In these studies, datasets are usually divided into training and test sets in a 7-to-3 ratio, allowing GBDT regression models to reach an average coefficient of determination of 0.953 for concentration prediction. A similar approach was applied in a portable electrochemical system for glyphosate detection, where a Bagged Trees binary classifier was trained using min–max current responses obtained from chronoamperograms, and its accuracy was evaluated through fivefold cross validation on datasets containing 30 samples per produce type to categorize results as safe and unsafe [31]. In contrast, ambient ionization mass spectrometry systems used ML for tracing the geographical origin of corn, with quantitative ion peak area data from differential exogenous pesticide analytes serving as features [2]. The RF model achieved 100% accuracy in the training set and 98.1% in external validation. Electronic nose applications for identifying insecticide residues on Swiss chard relied on raw sensor responses to volatile organic compounds, analyzed using PCA followed by a supervised SVM classifier [27]. This method, evaluated with fivefold cross validation, achieved a classification accuracy of about 92.3% across the tested samples.

### 4.8. Problem-Oriented Framework for Machine-Learning-Based Pesticide Analysis

**Figure 7:**
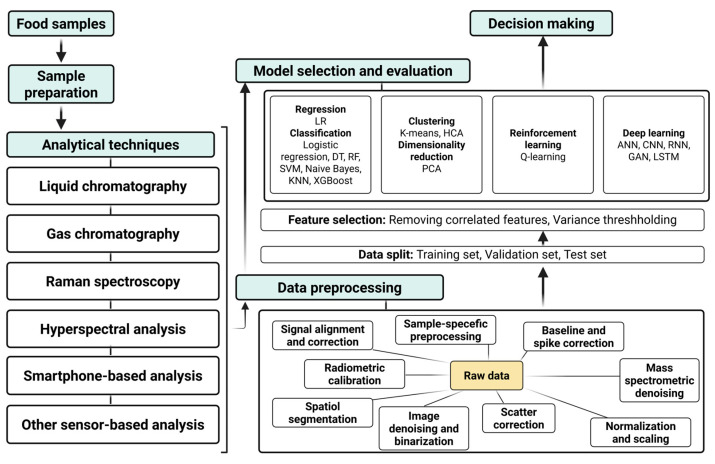
Problem-oriented workflow for machine-learning-assisted pesticide analysis in food samples.

Figure 7 shows a schematic workflow of ML applications in the pesticide analysis of food samples. The framework summarizes the main analytical steps, beginning with food sampling and sample preparation, followed by data acquisition using chromatographic, spectroscopic, hyperspectral, smartphone-based, and other sensor-based techniques. It then describes data preprocessing, feature selection, model construction, and the final decision stage. As illustrated, the selection of ML methods depends on the analytical goal. Tasks such as qualitative classification and quantitative determination, as well as high-accuracy laboratory analysis compared with rapid or on-site screening, require different data handling and modeling strategies. Classical regression and classification methods are commonly applied to structured chromatographic and spectral data, whereas clustering, dimensionality reduction, and DL approaches are better suited for high-dimensional or image-based data.

## 5. Perspectives

Future research should aim to improve the generalization and robustness of ML models by including a wider range of crop varieties and a broader spectrum of pesticide compounds across different concentration levels. One promising direction is the development of information fusion approaches, particularly the combination of spatial image data and spectral information in hyperspectral imaging systems, to enhance the predictive accuracy of non-destructive models. In addition, multi-modal analytical platforms, such as the integration of micro-hyperspectral methods with fluorescence data, may provide a more complete understanding of pesticide residue behavior in different food matrices. For food traceability applications, moving beyond purely non-target screening toward approaches that combine known external targets with efficient classification algorithms, such as RF, is likely to improve both the speed and accuracy of origin identification. Overall, the advancement of high-throughput systems and biomimetic sensors, together with modern DL architectures, is essential for the wider monitoring of agricultural products and for strengthening public health protection and global food safety.

## 6. Conclusions and Limitations

The use of machine learning and deep learning methods for pesticide residue analysis in food matrices shows high potential, although several challenges remain. Many machine learning models require large, diverse, and high-quality datasets for effective training and validation. However, such datasets are often difficult to obtain because of data ownership restrictions, limited access to analytical results, inconsistent experimental protocols, and the lack of openly available spectral and chromatographic libraries. Limited dataset diversity can reduce model generalizability and lead to lower performance when models are applied to new food matrices or variable real-world conditions. To address these limitations, there is a need for well-curated, standardized, and openly accessible databases that support reproducible and transferable machine learning applications.

Further challenges are linked to the complexity and variability of food matrices. Non-destructive spectroscopic techniques, such as hyperspectral imaging and surface-enhanced Raman spectroscopy, often face signal overlap, matrix interference, and reduced sensitivity at pesticide concentrations below ppm levels. Differences in food composition, processing methods, and storage conditions, along with variations in sample preparation, instrument settings, and data acquisition procedures, further affect model robustness and limit comparability between studies. The adoption of standardized analytical workflows, reference materials, and reporting guidelines is therefore essential to improve consistency and reliability.

Despite these constraints, the reviewed studies demonstrate the strong promise of machine-learning-based analytical systems. The combination of machine learning models, including convolutional neural networks, random forest algorithms, and ensemble methods, with fast and non-destructive sensing techniques, has achieved high performance in both the qualitative classification and quantitative prediction of pesticide residues and complex mixtures. As data availability expands, data-sharing practices improve and models are optimized to better reflect real-world variability. Machine learning is expected to play an important role in providing automated and scalable solutions for pesticide residue analysis, thereby supporting food safety control and regulatory compliance.

## Figures and Tables

**Table 1:** Summary of ML models and experimental parameters in the analysis of pesticides in different food matrices.

Target Pesticides (*n*)	Sample	Analytical Equipment and Software	ML Model	Input Variables	Training Data Size (Training: Validation)	Accuracy of ML Model	Ref.
Gas chromatography							
3	Apple peel	GCxGC/TOF-MS	RF	Metabolomic features data	44 apples (35:9)	100%	[21]
2	Saffron	GC-FID	PLSR	Chromatographic peak areas	24 calibration samples	-	[50]
Liquid chromatography							
398	Strawberries	UHPLC, HRMS	XGBoost, RF, LightGBM, KNN	Chemical descriptors and fingerprints	398 pesticides (321:77)	90%	[24]
4	Lettuce leaves	UHPLC-PDA	KNNR, RF, XGBoost	Immersion method, solution conc., ultrasonic power, immersion time, initial pesticide conc., control conc., pesticide type	Dataset from multiple immersion	98%	[33]
32	Pork and aquatic products	UHPLC, HRMS	ANN, RF, SVM, XGBoost	MS^2^ spectra intensity vectors	10,246 MS spectra (811:32)	86.9%	[51]
9	Corn kernels	LC–MS/MS	RF, SVM, AdaBoost, Gradient Boosting	Normalized ion intensities	120 validation samples	98.22%	[2]
823	Foods and vegetables	UHPLC–Q-Orbitrap MS	QSRR	SMILES strings, graph-derived topological descriptors, correlation weight features log tR	823 compounds	92%	[4]
Raman spectroscopy							
14	Cucumber, green pepper, and wheat flour	Custom-built and commercial Raman spectrometer	RF, PCA, HCA	Raman spectra	320 spectra total (70:30)	100%	[3]
3	Milk	Portable Raman spectrometer	CNN, RF, PCA–SVM, PCA–SVR	Raman spectra	400 spectra	99.25%	[12]
8	Apple pericarp	SERS	CNN, NB, LDA, KNN, SVM, DT, RF	Normalized SERS spectra	6000 spectra	99.62%	[28]
8	Apples	Raman microscope	PLS-DA, SVM, 1D-CNN	Raman spectra	2400 spectra	64–100%	[6]
1	Kale and spinach leaves	Portable Raman spectrometer, R	RF, SVM	Raman spectral data	2000 spectra (1600:400)	97%	[52]
Hyperspectral analysis							
6	Grape	HSI systems, MATLAB, Python	LR, SVM, RF, CNN, ResNet	Reflectance spectra	1071 clusters	93–98.8%	[19]
2	Lettuce leaves	Hyperspectral imaging	LSSVR with RBF kernel	SNV-preprocessed hyperspectral reflectance	200 samples (132:68)	88.9–93.9%	[53]
4	Hami melon surfaces	Hyperspectral camera	1D-CNN, KNN, RF	Hyperspectral reflectance spectra	1600 spectra	94.0%	[30]
1	Sorghum grains	Hyperspectral camera	GBDT, XGBoost, LGBM, CatBoost	Reflectance spectra	2139 spectra	98%	[11]
1	Tea chrysanthemum leaves	Hyperspectral spectroradiometer, MATLAB, Python	PLSR, SVR	Hyperspectral reflectance data	124 total samples (93:31)	65.4%	[54]
Smartphone-based analysis							
1	Rice, millet, and ginger	Smartphone imaging	FNN, DT, SVR, Ridge Regression, Bayesian Regression	RGB values extracted from fluorescence images	70% training, 15% validation, 15% test	99.5%	[34]
5	Fruits and vegetables	Smartphone imaging, MATLAB	GP–SR, LR	RGB color features	70% training, 30% testing split	-	[1]
1	Apples, lettuce	Smartphone with UV lamp for fluorescence	KNN	Fluorescence signal values	90 samples total (72:18)	100%	[55]
Spectrophotometer-based analysis							
2	Red wine	Lab-built spectrophotometer	SVR, DT, NN	Fluorescence spectra	24 calibration, 12 tests; 27 calibration, 3 wine brands for testing	-	[5]
3	Kumquat (*Citrus japonica*) fruit surfaces	Lab-built spectrophotometer	SPA–SVM, 1D–CNN, 1D–ResNet	Preprocessed spectra	3146 spectra	97%	[20]
1	Cabbage juice	Fluorescence spectroscopy	KNN, DT, PLSR, ESN	Fluorescence spectral intensities	130 samples	97%	[26]
3	Apple juice	UV–Vis spectrophotometer	SSA–BPNN, BPNN, PSO–BPNN, ELM	UV spectral data	84 samples (56:28)	99.9%	[7]
7	Tea	SERS, SEM, UV–Vis, XRD	KNN, DT, RF, GBDT, SVC, dimensionality reduction via t-SNE, PCA, MDS, PLS	Normalized SERS spectra	600 spectra	99.72%	[32]
4	Spinach	UV–Vis, MATLAB, Origin	Bayesian-optimized Random Forest	Activity-change signals	1040 samples	100%	[56]
Other sensor-based analysis							
1	Apples, strawberries, bell peppers and carrots	ElectrochemSENSE portable electrochemical immunosensor	BTC	Current response	150 total samples	86.4%	[25]
1	Wild chard leaves	Homemade electronic nose	PCA + SVM	3 features per sensor (max value, area, stabilized value)	40 samples	96.5%	[27]
3	Tea	Electrochemical biosensor platform with heating thermostatic screen-printed electrode, GC	LDA–RF, GBDT	Time-series impedance data	270 samples per pesticide	96.3%	[31]

**Table 2:** Evaluation metrics for machine learning classification models in pesticide residue analysis. The confusion matrix tallies True Positives (TP), True Negatives (TN), False Positives (FP), and False Negatives (FN).

Metric	Formula	Interpretation	Use Case
Accuracy	$\frac{\mathrm{TP}+\mathrm{TN}}{\mathrm{Total}}$	Overall correctness	Only useful for balanced datasets. In a 99:1 imbalance, a “dummy” model predicting the majority class achieves 99% accuracy
Precision	$\frac{\mathrm{TP}}{\mathrm{TP}+\mathrm{FP}}$	Positive predictive value. How many predicted positives are actually positive?	Critical when False Positives are costly
Recall	$\frac{\mathrm{TP}}{\mathrm{TP}+\mathrm{FN}}$	How many actual positives did we capture?	Critical when false negatives are dangerous
F1 Score	$2\times \frac{P\times R}{P+R}$	Harmonic means of Precision and Recall	Best single metric for imbalanced data where both error types of matter. Penalizes extreme values

## Data Availability

No new data were created or analyzed in this study. Data sharing is not applicable to this article.

## Abbreviations

The following abbreviations are used in this manuscript:

1D–CNN	One-dimensional Convolutional Neural Network
1D–ResNet	One-dimensional Residual Network
ANN	Artificial Neural Network
BPNN	Back Propagation Neural Network
BTC	Bagged Tree Classifier
CatBoost	Categorical Boosting
CNN	Convolutional Neural Network
DL	Deep Learning
DT	Decision Tree
ELM	Extreme Learning Machines
ESN	Echo State Network
FNN	Feedforward Neural Network
GBDT	Gradient-Boosting Decision Tree
GC-FID	Gas Chromatography–Flame Ionization Detector
GC-MS	Gas Chromatography–Mass Spectrometry
GCxGC-MS	Two-Dimensional Gas Chromatography–Mass Spectrometry
GP–SR	Improved Genetic Programming–Symbolic Regression
HCA	Hierarchical Clustering Analysis
HPLC	High-Performance Liquid Chromatography
HSI	Hyperspectral Imaging
KNN	K-Nearest Neighbor
KNNR	K-Nearest Neighbor Resampling
LDA	Linear Discriminant Analysis
LDA–RF	Linear Discriminant Analysis–Random Forest
LC	Liquid Chromatography
LC-HRMS	Liquid Chromatography High-Resolution Mass Spectrometry
LightGBM	Light Gradient-Boosting Machine
LR	Linear Regression
LSSVR	Least Squares Support Vector Regression
MDS	Multidimensional Scaling
ML	Machine Learning
MRL	Maximum Residue Limit
NN	Neural Network
PCA	Principal Component Analysis
PLS	Partial Least Squares
PLSR	Partial Least Squares Regression
PSO–BPNN	Particle Swarm Optimization–Backpropagation Neural Network
QSRR	Quantitative Structure–Retention Relationship
RBF	Radial Basis Function
RBM	Restricted Boltzmann Machines
RF	Random Forest
ResNet	Residual Network
RMSE	Root Mean Square Error
RT	Retention Time
SERS	Surface-Enhanced Raman Scattering
SSA–BPNN	Sparrow Search Algorithm–Back Propagation Neural Network
SVC	Support Vector Classification
SVM	Support Vector Machine
SVMR	Support Vector Machine Regression
SVR	Support Vector Regression
t-SNE	t-Distributed Stochastic Neighbor Embedding
UV	Ultraviolet
VNIR	Visible Near-Infrared
XGBoost	Extreme Gradient Boosting

## References

[1] Dai J., Chen X., Zhang Y., Zhang M., Dong Y., Zheng Q., Liao J., Zhao Y. (2025). Machine Learning-Enhanced Color Recognition of Test Strips for Rapid Pesticide Residue Detection in Fruits and Vegetables. Food Control.

[2] Su H., Han L., Ge M., Wang X., Zeng H., Zhao D., Xiong W., Wen L. (2025). Simultaneous Determination of Pesticide Residues and Rapid Discrimination of Corn Production Origin Using Ambient Ionization Mass Spectrometry Combined with Machine Learning. Food Chem..

[3] Yüce M., Öncer N., Çınar C.D., Günaydın B.N., Akçora Z.İ., Kurt H., Yüce M., Öncer N., Çınar C.D., Günaydın B.N. (2025). Comprehensive Raman Fingerprinting and Machine Learning-Based Classification of 14 Pesticides Using a 785 Nm Custom Raman Instrument. Biosensors.

[4] Lotfi S., Ahmadi S., Azimi A., Toropova A.P., Toropov A.A. (2025). In Silico Prediction of Pesticide Residue Retention Times in Foods and Vegetables Using the Monte Carlo Technique. Food Res. Int..

[5] He J.-R., Wei J.-W., Chen S.-Y., Li N., Zhong X.-D., Li Y.-Q., He J.-R., Wei J.-W., Chen S.-Y., Li N. (2022). Machine Learning-Assisted Synchronous Fluorescence Sensing Approach for Rapid and Simultaneous Quantification of Thiabendazole and Fuberidazole in Red Wine. Sensors.

[6] Castillo-Girones S., Arnould Q., Gómez-Sanchis J., Blasco J., Pigeon O., Baeten V., Fernández Pierna J.A. (2025). Raman Spectroscopy for Multi-Label Identification of Common Apple Pesticide Mixtures Using CNNs and Gradient-Weighted Class Activation Mapping. Food Control.

[7] Meng D., Yu X., Xu L., Zhang W., Zhao Z. (2025). Simultaneous Detection of Imidacloprid, Acetamiprid, and Clothianidin in Apple Juice Using Ultraviolet Spectroscopy and the SSA–BPNN Model. J. Appl. Spectrosc..

[8] Alimzhanova M., Meirbekov N., Syrgabek Y., López-Serna R., Yegemova S. (2025). Plant- and Microbial-Based Organic Disease Management for Grapevines: A Review. Agriculture.

[9] Ahmad M.F., Ahmad F.A., Alsayegh A.A., Zeyaullah M., AlShahrani A.M., Muzammil K., Saati A.A., Wahab S., Elbendary E.Y., Kambal N. (2024). Pesticides Impacts on Human Health and the Environment with Their Mechanisms of Action and Possible Countermeasures. Heliyon.

[10] Li Z., Fantke P. (2022). Toward Harmonizing Global Pesticide Regulations for Surface Freshwaters in Support of Protecting Human Health. J. Environ. Manag..

[11] Peng J., Zhang J., Han L., Ma X., Hu X., Lin T., He L., Yi X., Tian J., Chen M. (2024). Determination of Malathion Content in Sorghum Grains Using Hyperspectral Imaging Technology Combined with Stacked Machine Learning Models. J. Food Compos. Anal..

[12] Li H., Hasi W., Li N., Liu X., Fang G. (2026). Machine Learning-Enhanced SERS Detection of Melamine and Its Analogues in Non-Pretreated Milk via Filter-Pressing Assembled Polytetrafluoroethylene-AgNPs Substrate. Spectrochim. Acta Part A Mol. Biomol. Spectrosc..

[13] Lebanov L., Tedone L., Ghiasvand A., Paull B. (2020). Random Forests Machine Learning Applied to Gas Chromatography—Mass Spectrometry Derived Average Mass Spectrum Data Sets for Classification and Characterisation of Essential Oils. Talanta.

[14] Matyushin D.D., Buryak A.K. (2020). Gas Chromatographic Retention Index Prediction Using Multimodal Machine Learning. IEEE Access.

[15] Syrgabek Y., Alimzhanova M. (2022). Modern Analytical Methods for the Analysis of Pesticides in Grapes: A Review. Foods.

[16] Choi E., Yoo W.J., Jang H.-Y., Kim T.-Y., Lee S.K., Oh H.B. (2023). Machine Learning Liquid Chromatography Retention Time Prediction Model Augments the Dansylation Strategy for Metabolite Analysis of Urine Samples. J. Chromatogr. A.

[17] Turova P., Stavrianidi A., Svekolkin V., Lyskov D., Podolskiy I., Rodin I., Shpigun O., Buryak A., Turova P., Stavrianidi A. (2022). Analysis of Primary Liquid Chromatography Mass Spectrometry Data by Neural Networks for Plant Samples Classification. Metabolites.

[18] Fuente-Ballesteros A., Tian L., Liu L., Ares A.M., Bayen S., Bernal J. (2025). Development and Validation of a Green and Practical Method for Studying Pesticides and Related Chemical Compounds in Bee Pollen Samples by UAE-LC-QTOF-MS. J. Food Compos. Anal..

[19] Ye W., Yan T., Zhang C., Duan L., Chen W., Song H., Zhang Y., Xu W., Gao P., Ye W. (2022). Detection of Pesticide Residue Level in Grape Using Hyperspectral Imaging with Machine Learning. Foods.

[20] Dai Q., Luo Z., Li Z., Lyu S., Xue X., Song S., Yu S., Huang Y., Dai Q., Luo Z. (2025). Field-Based, Non-Destructive, and Rapid Detection of Pesticide Residues on Kumquat (*Citrus japonica*) Surfaces Using Handheld Spectrometer and 1D-ResNet. Agronomy.

[21] Barberis E., Amede E., Dondero F., Marengo E., Manfredi M., Barberis E., Amede E., Dondero F., Marengo E., Manfredi M. (2021). New Non-Invasive Method for the Authentication of Apple Cultivars. Foods.

[22] Syrgabek Y., Alimzhanova M., Yegemova S., Batyrbekova S. (2024). Vacuum-Assisted Headspace-Solid Phase Microextraction of Pesticides in Grape Samples. Adv. Sample Prep..

[23] Alygizakis N., Konstantakos V., Bouziotopoulos G., Kormentzas E., Slobodnik J., Thomaidis N.S., Alygizakis N., Konstantakos V., Bouziotopoulos G., Kormentzas E. (2022). A Multi-Label Classifier for Predicting the Most Appropriate Instrumental Method for the Analysis of Contaminants of Emerging Concern. Metabolites.

[24] Feng C., Xu Q., Qiu X., Jin Y., Ji J., Lin Y., Le S., She J., Lu D., Wang G. (2021). Evaluation and Application of Machine Learning-Based Retention Time Prediction for Suspect Screening of Pesticides and Pesticide Transformation Products in LC-HRMS. Chemosphere.

[25] Dhamu V.N., Prasad S. (2020). ElectrochemSENSE: A Platform towards Field Deployable Direct on-Produce Glyphosate Detection. Biosens. Bioelectron..

[26] Wu D., Sun X., Liu Y., Liu C., Wu J. (2025). The Fluorescence Spectrum Combined with a Broad Learning System to Characterize the Content of Difenoconazole in Cabbage. Anal. Methods.

[27] Amkor A., Aboulkacem A., Barbri N.E. (2023). An Electronic Nose for Insecticides Detection in Food: The Case of Alpha-Cypermethrin in Swiss Chard. Bull. Electr. Eng. Inform..

[28] Wang X., Jiang S., Liu Z., Sun X., Zhang Z., Quan X., Zhang T., Kong W., Yang X., Li Y. (2024). Integrated Surface-Enhanced Raman Spectroscopy and Convolutional Neural Network for Quantitative and Qualitative Analysis of Pesticide Residues on Pericarp. Food Chem..

[29] Saha D., Manickavasagan A. (2021). Machine Learning Techniques for Analysis of Hyperspectral Images to Determine Quality of Food Products: A Review. Curr. Res. Food Sci..

[30] Hu Y., Ma B., Wang H., Zhang Y., Li Y., Yu G. (2023). Detecting Different Pesticide Residues on Hami Melon Surface Using Hyperspectral Imaging Combined with 1D-CNN and Information Fusion. Front. Plant Sci..

[31] Yang N., Tao S., Mao H., Wei M., Fu J., Song W. (2024). An Integrated Platform and Method for Rapid High-Throughput Quantitative Detection of Organophosphorus Pesticide Residues. IEEE Trans. Instrum. Meas..

[32] Li S., Cao T., Xu M., He M., Cai X., Lu Y., Jin Y., Han M., Liu H., Wang C. (2025). Biomimetic SERS Platform with Machine Learning for Sensitive Detection of Pesticides in Complex Food Matrices. Opt. Laser Technol..

[33] Zhao S., Huang X., Chen G., Qin H., Xu B., Luo Y., Liao Y., Wang S., Yan S., Zhao J. (2024). Causal Inference and Mechanism for Unraveling the Removal of Four Pesticides from Lettuce (*Lactuca sativa* L.) via Ultrasonic Processing and Various Immersion Solutions. Ultrason. Sonochemistry.

[34] Cheng Z., Liu X., Li R., Liu X., Zhang X., Feng X., Zhou L. (2025). A Fluorescence Probe-Smartphone-Machine Learning Integrated Platform for the Visual and Intelligent Detection of Imidacloprid in Agricultural Products. Food Chem..

[35] González-Domínguez R., Sayago A., Fernández-Recamales Á., González-Domínguez R., Sayago A., Fernández-Recamales Á. (2022). An Overview on the Application of Chemometrics Tools in Food Authenticity and Traceability. Foods.

[36] Patiyal A., Pandit D., Mushtaq M. (2026). A Systematic Review of Recent Developments and Trends in Fungicide Residue Analysis in Agriculture Crops. Microchem. J..

[37] Li J., Wu A., Liu L., Qu A., Xu C., Kuang H., Xu L. (2025). Analysis of Food Safety Based on Machine Learning: A Comprehensive Review and Future Prospects. Food Chem..

[38] Li J., Qian J., Chen J., Ruiz-Garcia L., Dong C., Chen Q., Liu Z., Xiao P., Zhao Z. (2025). Recent Advances of Machine Learning in the Geographical Origin Traceability of Food and Agro-Products: A Review. Compr. Rev. Food Sci. Food Saf..

[39] Zhang Q., Lu Z., Liu Z., Li J., Chang M., Zuo M., Zhang Q., Lu Z., Liu Z., Li J. (2025). Application of Machine Learning in Food Safety Risk Assessment. Foods.

[40] Adade S.Y.-S.S., Lin H., Johnson N.A.N., Nunekpeku X., Aheto J.H., Ekumah J.-N., Kwadzokpui B.A., Teye E., Ahmad W., Chen Q. (2025). Advanced Food Contaminant Detection through Multi-Source Data Fusion: Strategies, Applications, and Future Perspectives. Trends Food Sci. Technol..

[41] Rodionova O.Y., Oliveri P., Malegori C., Pomerantsev A.L. (2024). Chemometrics as an Efficient Tool for Food Authentication: Golden Pillars for Building Reliable Models. Trends Food Sci. Technol..

[42] Pai D.G., Balachandra M., Kamath R. (2025). Explainable AI in Agriculture: Review of Applications, Methodologies, and Future Directions. Eng. Res. Express.

[43] Pandey V.K., Srivastava S., Dash K.K., Singh R., Mukarram S.A., Kovács B., Harsányi E., Pandey V.K., Srivastava S., Dash K.K. (2023). Machine Learning Algorithms and Fundamentals as Emerging Safety Tools in Preservation of Fruits and Vegetables: A Review. Processes.

[44] Chakraborty P., Borras E., Rajapakse M.Y., McCartney M.M., Bustamante M., Mitcham E.J., Davis C.E. (2023). Non-Destructive Method to Classify Walnut Kernel Freshness from Volatile Organic Compound (VOC) Emissions Using Gas Chromatography-Differential Mobility Spectrometry (GC-DMS) and Machine Learning Analysis. Appl. Food Res..

[45] Shi Y.-F., Yang Z.-X., Ma S., Kang P.-L., Shang C., Hu P., Liu Z.-P. (2023). Machine Learning for Chemistry: Basics and Applications. Engineering.

[46] Olszewski S., Lurie I., Lytvynenko V., Demchenko V., Kornilovska N., Boskin O. Regression Modeling for Monitoring Organochlorine Pesticide Residues. https://ceur-ws.org/Vol-3426/paper17.pdf.

[47] Kobayashi Y., Yoshida K. (2022). Automated Retention Time Prediction of New Psychoactive Substances in Gas Chromatography. Procedia Comput. Sci..

[48] Fedorova E.S., Matyushin D.D., Plyushchenko I.V., Stavrianidi A.N., Buryak A.K. (2022). Deep Learning for Retention Time Prediction in Reversed-Phase Liquid Chromatography. J. Chromatogr. A.

[49] Chang Y.-T., Hsueh M.-C., Hung S.-P., Lu J.-M., Peng J.-H., Chen S.-F. (2021). Prediction of Specialty Coffee Flavors Based on Near-Infrared Spectra Using Machine- and Deep-Learning Methods. J. Sci. Food Agric..

[50] Zarinkhat F., Parastar H. (2025). Chemometrics-Assisted Liquid-Phase Microextraction Combined with Gas Chromatography for Determination of Ametryn and Chlorpyrifos in Saffron. Microchem. J..

[51] Chen T., Liang W., Zhang X., Wang Y., Lu X., Zhang Y., Zhang Z., You L., Liu X., Zhao C. (2024). Screening and Identification of Unknown Chemical Contaminants in Food Based on Liquid Chromatography–High-Resolution Mass Spectrometry and Machine Learning. Anal. Chim. Acta.

[52] Thuku J.M., Kaniu M.I., Ndung’u C.N., Kiruri L.W., Kaduki K.A. (2025). Rapid Trace Detection of Chlorpyrifos in Vegetables Using 2D Raman Correlation Spectroscopy and Machine Learning. LWT.

[53] Sun J., Cong S., Mao H., Wu X., Yang N. (2018). Quantitative Detection of Mixed Pesticide Residue of Lettuce Leaves Based on Hyperspectral Technique. J. Food Process Eng..

[54] Lu J., Zhang Q., Qi Q., Zheng G., Zhang J., Chen S., Zhang F., Fang W., Guan Z., Chen F. (2025). Machine Learning-Assisted Hyperspectral Reflectance Analysis for Non-Destructive Detection of Clothianidin Residues in Tea Chrysanthemum. Microchem. J..

[55] Wang X., Cao Z., Shi B., Zhao A., Dai J., Wu S.-T., Wang D., Liu W. (2025). Machine Learning-Assisted Photoelectrochemical/Fluorescence Dual-Mode Sensor for Ultra-Sensitive Glyphosate Detection. Chem. Eng. J..

[56] Song D., Cheng Y., Hu Y., Ge K., Fan J., Shi X., Huang H., Li Y. (2026). Machine Learning-Assisted Multi-Channel Nanozyme Sensor Arrays for Multiple Pesticide Tracking, Tracing and Metabolism Analysis. Biosens. Bioelectron..

[57] Gaida M., Cain C.N., Synovec R.E., Focant J.-F., Stefanuto P.-H. (2023). Tile-Based Random Forest Analysis for Analyte Discovery in Balanced and Unbalanced GC × GC-TOFMS Data Sets. Anal. Chem..

[58] Jiménez-Carvelo A.M., González-Casado A., Bagur-González M.G., Cuadros-Rodríguez L. (2019). Alternative Data Mining/Machine Learning Methods for the Analytical Evaluation of Food Quality and Authenticity—A Review. Food Res. Int..

[59] Syrgabek Y., Alimzhanova M., Haque S.M., Psillakis E., Fuente-Ballesteros A. (2026). Vacuum-Assisted Headspace Solid-Phase Microextraction in Food Analysis: Basics and Applications. Anal. Chim. Acta.

[60] Alimzhanova M., Syrgabek Y., Garcia Encina P.A. (2025). Eco-Friendly Miniaturized Solid Phase Microextraction for Pesticide Analysis in Grapes. J. Sep. Sci..

